# The Effect of Intravenous Infusions of Glutamine on Duodenal Cell Autophagy and Apoptosis in Early-Weaned Calves

**DOI:** 10.3390/ani9070404

**Published:** 2019-07-01

**Authors:** Xusheng Dong, Ruina Zhai, Zhaolin Liu, Xueyan Lin, Zhonghua Wang, Zhiyong Hu

**Affiliations:** 1 Ruminant Nutrition and Physiology Laboratory, College of Animal Science and Technology, Shandong Agricultural University, Taian 271018, China; 2 College of Animal Science, Xinjiang Agricultural University, Urumqi 830052, China

**Keywords:** calf, glutamine, autophagy, apoptosis

## Abstract

**Simple Summary:**

The objective of this study was to determine the effects of intravenous infusions of L-glutamine (Gln) on the autophagy and apoptosis of duodenum cells in weaned calves. The results showed that the autophagy level of duodenal cells was increased with an increasing Gln infusion dose (0 to 20 g/d) and dropped when Gln was further increased to 40 g/d. We also found that the level of apoptosis was decreased with an increasing Gln infusion dose from 0 to 20 g/d, and then rose as the dose increased to 40 g/d. This knowledge will provide a reference for weaned calf health management.

**Abstract:**

The objectives of this study were to determine the effects of intravenous infusions of L-glutamine (Gln) on the autophagy and apoptosis of duodenum cells in early-weaned calves. Holstein male calves were weaned at day 35 (20 male calves, birth weight 43 ± 1.8 kg; 35 ± 3 d of age) and randomly allocated to four treatments (5 calves/treatment). The treatments were: (1) infusion of NaCl, representing the control group (C); (2) infusion of 10 g/d of Gln solution (L); (3) infusion of 20 g/d of Gln solution (M); and (4) infusion of 40 g/d of Gln solution (H). The solutions were infused for 2 h daily for 3 consecutive days after weaning. All calves were killed on the third day post-weaning. The results showed that the autophagy level of the duodenal cells was increased as the Gln infusions increased from 0 to 20 g/d and dropped with a further increase in dose (40 g/d). We also found that the level of apoptosis was decreased with Gln infusion from 0 to 20 g/d and rose as the dose increased to 40 g/d. This knowledge provides a reference for weaned calf health management.

## 1. Introduction

Glutamine (Gln) is the most abundant amino acid in vivo and is a major respiratory fuel and metabolic precursor for many cell types [1]. Glutamine, which once was regarded as a nonessential amino acid, has recently been termed conditionally essential during injury or oxidative stress [2,3]. A previous study suggested that Gln could protect the small intestine from various harmful injuries in rats [4]. Kallweit et al. [5] showed that Gln protects intestinal cells from both heat and oxidant stress. Recently, researchers attempted to evaluate the impact of Gln on autophagy and apoptosis [6,7,8]. Sakiyama et al. [7] suggested that Gln could protect intestinal epithelial cells by enhancing autophagy.

Autophagy is a specific protein degradation process that functions in the bulk degradation of cellular components and has been recognized as an important mechanism for cell survival under conditions of stress [7,9]. When cells lack nutrients, autophagy is activated to supply amino acids in order to maintain cell survival [10]. In vivo apoptosis and autophagy are two forms of physiological and conserved programmed cell death [11]. Apoptosis is characterized by a series of morphological changes, including plasma membrane blebbing, nuclear condensation, and fragmentation, which lead to the formation of apoptotic bodies [12]. When cells are under stress, autophagy and apoptosis are activated [12]. In general, autophagy is activated first and maintains cell homeostasis [13]. When stress is prolonged or exceeds a threshold, apoptosis is activated [12,14]. The protein microtubule-associated protein 1 light chain 3-Ⅱ(LC3-Ⅱ), which is a useful marker of autophagic membranes, is essential for the expansion of the early autophagosome in the context of cellular house-keeping and autophagic cell death [15,16]. Caspase-3 is an executioner caspase, which is activated by apoptosis [12]. Furthermore, PI3K/Akt/mTOR signaling pathways, which inhibit autophagy, have been found to be essential for the regulation of autophagy [17]. A previous study found that caffeine could induce autophagy by abolishing AKT phosphorylation [17]. The kinase mammalian target of rapamycin (mTOR) is a downstream target of the PI3K/AKT pathway [17]. Deactivation of mTOR signaling induces autophagy [18]. Amino acids, which are provided by autophagy, can restore mTOR complex 1 (mTORC1) activity during amino acid starvation [8]. The restoration of mTORC1 in turn inhibits autophagy, which completes the feedback loop [8]. The feedback loop could protect the cells by mitigating damage from stress and starvation.

Weaning is the transition from the ingestion of milk to solid feed for calves with dramatic gastrointestinal transformations [19]. Weaning is a particularly vulnerable period for mammals, with an increased risk of malnutrition, intestinal infections, and poor growth [20,21]. The function and morphology of the small intestine are severely disturbed after weaning, such as villous shortening in pigs [22,23]. Our previous study found that an exogenous supply of Gln increased the autophagy level of liver cells and increased growth rates, villus height, and crypt depth of the duodenum in early-weaned calves [20,24]. Whether Gln induces autophagy and apoptosis of the duodenum cells in early-weaned calves remains unknown. The purpose of this study was to evaluate the effect of Gln on the autophagy and apoptosis of duodenum cells in early-weaned calves. We hypothesized that intravenous infusions of Gln would increase the level of autophagy and reduce the level of apoptosis. This would provide a reference for weaned calf health management.

## 2. Materials and Methods

Animal care and use were approved and conducted under established standards of the Ethics Committee on animals of Shandong Agricultural University (SDAUA-2018-012). The study was conducted during November of 2018 at the Shandong high-speed modern dairy farm in Ji Nan, Shandong, China. The animals were individually housed in a pen with free access to water and fresh calf starter. The ingredient and nutrient composition of the calf starter is given in Table 1.

All calves received 4 L of colostrum in the 2-hour period after birth and were then fed 6 L of whole milk 3 times daily until weaning. Fresh calf starter was offered ad libitum beginning at 3 d of age. Water was offered daily ad libitum. Holstein calves were weaned at day 35 (20 male calves, birth weight 43 ± 1.8 kg; 35 ± 3 d of age) and randomly allocated to four treatments (5 calves/treatment). Starting from day 35, the calves were given the following treatments for 3 consecutive days. The treatments were: (1) infusion of 1.5 L of 0.85% NaCl, representing the control group (C); (2) infusion of 10 g/d of Gln mixed with 1.5 L of 0.85% NaCl solution (L); (3) infusion of 20 g/d of Gln mixed with 1.5 L of 0.85% NaCl solution (M); and (4) infusion of 40 g/d of Gln mixed with 1.5 L of 0.85% NaCl solution (H). The dose of intravenous infusion Gln referred to that in a previous study [20]. At the beginning of the experiment, all calves had milk removed from their daily diet. The solutions were infused for 2 h daily for 3 consecutive days after weaning. Starter intake for each calf was measured daily during the infusion period.

All calves were euthanized following captive bolt gun stunning on the 3 d post-weaning day for measuring the autophagy and apoptosis of duodenum cells. After opening the body cavity, the samples of duodenum (entire wall from 6 cm distal to the pylorus) were immediately frozen in liquid nitrogen and stored at −80 °C until western blotting was performed. 

Briefly, the tissue sample blocks (entire duodenum from 6 cm distal to the pylorus) were washed with phosphate buffer saline (PBS, Solarbio, P1020-500 mL, Beijing, China), cut into small pieces, homogenized in PBS at 4 °C using a Servicebio KZ-Ⅱ homogenizer, kept on ice for 0.5 h, oscillated to ensure complete tissue cracking every 5 min, and then centrifuged (3000× g, 10 min, 4 °C). Protein concentration was determined in the supernatant (BCA Protein Assay Kit, G2026, Servicebio, Wuhan, China). The sample was then diluted with an equal volume of Laemmli sample buffer (Bio-Rad, 1610737, Shanghai, China) and boiled for 5 min. Sodium dodecyl sulfate-PAGE, electro-transfer of proteins, and immunoblotting were performed as previously described [25,26]. Antibodies used for immunoblotting were anti-LC3 (Sigma-Aldrich, L8918, Shanghai, China), anti-Caspase-3 (Sigma-Aldrich, C8487, Shanghai, China), anti-mTOR (Sigma-Aldrich, SAB2701843, Shanghai, China), anti-phospho-mTOR (Sigma-Aldrich, SAB4301526, Shanghai, China), anti-β-actin (Sigma-Aldrich, A2066, Shanghai, China), and appropriate secondary antibodies (Servicebio, GB23303, Wuhan, China). The chemiluminescence of bands of interest were detected with a digital G: Box imager (Syngene, Frederick, MD, USA). The band density was quantified with ImageJ software (National Institutes of Health, Bethesda, MD, USA). 

The data were analyzed as a completely randomized design using one-way ANOVA of SAS 8.2 (SAS Institute Inc., Cary, NC). The individual calf was considered as the experimental unit. The analysis used the following model: y*ij* = μ + α*i* + ε*ij* (y = western blot data, μ = mean, i = dose of infusions, and ε = residuals). The means were compared using Duncan’s multiple range test. Significance was declared at *p* < 0.05.

## 3. Results

**Figure 1:**
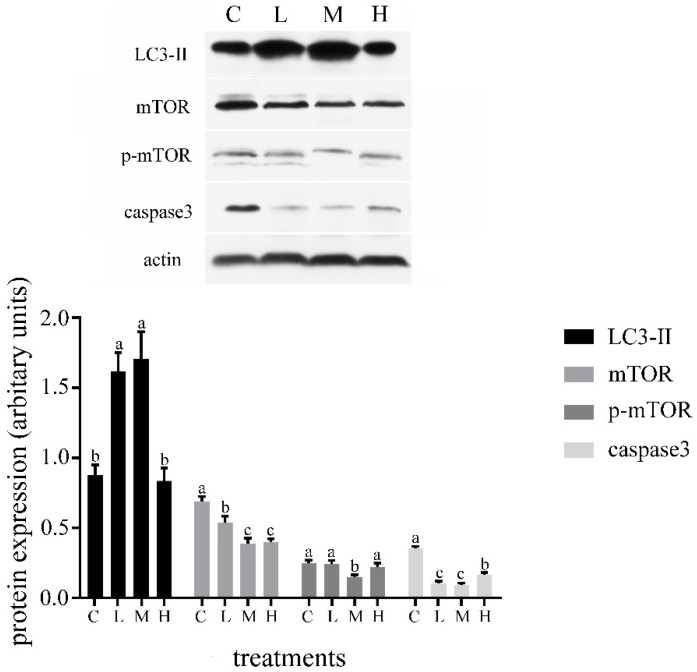
Effects of glutamine (Gln) infusions on the microtubule-associated protein 1 light chain 3-Ⅱ (LC3-Ⅱ), mTOR, p-mTOR, and caspase3 expression of duodenum in weaned calves. Treatment was as follows: (1) C: infusion of 1.5 L of 0.85% NaCl; (2) L: infusion of 10 g/d of Gln mixed with 1.5 L of 0.85% NaCl; (3) M: infusion of 20 g/d of Gln mixed with 1.5 L of 0.85% NaCl; (4) H: infusion of 40 g/d of Gln mixed with 1.5 L of 0.85% NaCl. Insets depict representative blots. Values represent means ± SD. Response from statistical result, *p* < 0.05. β-Actin was used to normalize the expression of target proteins. The letters below the bar graph indicate different treatments. Different letters above the bar indicate differences between different groups (*p* < 0.05).

The starter intake of group C, L, M, and H were 1.12 kg/d, 1.15 kg/d, 1.22 kg/d, and 1.19 kg/d, respectively. Starter intake was not different between treatments. The results reported in this research showed that the autophagy level of the duodenal cells was increased with an increasing Gln infusion dose (0 to 20 g/d) and dropped when Gln was further increased to 40 g/d (Figure 1). We also found that the level of apoptosis was decreased with an increasing Gln infusion dose from 0 to 20 g/d, and then rose with an increasing dose of Gln to 40 g/d (Figure 1). In group M, the level of autophagy reached the highest level; in contrast, the level of apoptosis reached a lowest point. The expression of mTOR was significantly decreased after Gln infusion (*p* < 0.05). The expression of p-mTOR in group M was lower than that in other groups (*p* < 0.05).

## 4. Discussion

In this study, we demonstrated that low dose infusion of Gln could induce autophagy and retard apoptosis. We further increased the infusion of Gln to 40 g/d and found that the effect of Gln on calves was reduced. We concluded that Gln-induced autophagy is mainly dependent on the inhibition of mTOR phosphorylation. Gln is involved in stress protection by way of the stimulation of autophagy in intestinal cells [7,27]. A previous study found that Gln infusion increased growth rates, villus height, and crypt depth in the duodenum of early-weaned calves [24]. In this study, when the concentration of Gln was increased from 0 to 20 g/d, the autophagy levels increased as the Gln infusion dose increased. The results suggested that Gln can promote autophagy in the duodenum. This finding is consistent with that of Sakiyama et al. [7], who confirmed that Gln is essential for maintaining autophagy and mounting an autophagic response under stress in intestinal cells. It has been suggested that Gln can induce autophagy in intestinal epithelial cells through restraining mTOR and p38 MAP kinase pathways [7]. The expression of mTOR and p-mTOR in our study is also consistent with this study. The expression of p-mTOR was significantly decreased after Gln infusion. To further investigate the effects of high dose Gln, we further increased the infusion of Gln to 40 g/d. Interestingly, we found that the autophagy level was decreased as the Gln infusion dose increased from 20 to 40 g/d. A possible explanation for this might be that the activity of Gln synthetase in the body gradually decreases when the blood concentration of Gln was excessive. In *Escherichia coli*, Gln synthetase activity is subject to inhibition by different end products of Gln metabolism [28]. A previous report suggested that over expression of Gln synthetase inhibited mTOR activity and activated autophagy [29]. Thus, the level of autophagy induced by Gln synthetase was decreased as the activity of Gln synthetase decreased.

Normally, autophagy restrains the activity of apoptosis, and apoptosis-associated caspase activation shuts off the autophagic process [12]. In our study, we also found that the level of apoptosis fell to a low point with the intravenous infusion dose of 20 g/d. The tendency of apoptosis was opposite to that of autophagy, which suggested that autophagy may inhibit the activity of apoptosis. Kallweit et al. [5] found that Gln protects intestinal cells from both heat and oxidant injury, which are key mechanisms in the prevention of apoptosis. Han et al. [30] demonstrated that the combination of Gln has the ability to maintain the integrity of the intestinal mucosal barrier by inhibiting the apoptosis of intestinal epithelial cells. These results are similar to our study. Our result suggests that the effect of Gln on apoptosis is contrary to that on autophagy. AKT is a kinase with dual autophagy–apoptosis regulatory potential, which can phosphorylate Beclin 1 and B-cell lymphoma-2 antagonists of cell death (BAD) to inhibit autophagic and apoptotic functions, respectively [12]. The activation of AKT could inhibit autophagy by inducing mTOR [12]. Thus, considerably more work needs to be done to determine the effect of Gln on Akt/mTOR signaling pathways in early-weaned calf.

In commercial dairy farms, dairy calves are weaned early to reduce milk costs. However, the gastrointestinal tract of the calf is not ready for early weaning [19]. In our research, intravenous infusions of low dose Gln could increases autophagy, which probably relieved weaning stress. A further study could assess the effects of diet supplement Gln on the early-weaned calf. This knowledge will provide a reference for Gln supplementation for weaned calf health management.

## 5. Conclusions

In conclusion, Gln could induce autophagy and decrease the level of apoptosis in the duodenum of early-weaned calves. The intravenous infusion moderate dose (20 g/d) of Gln is most effective. This knowledge will provide a reference for weaned calf health management.

## Figures and Tables

**Table 1:** Ingredient and nutrient composition of the experimental starter of calves.

Items	Content (% of DM)
Ingredients	
Corn grain	48
Wheat bran	12.6
Soybean meal	18.8
Extruded soybean	7
Corn gluten meal	9
Salt	0.55
Calcium carbonate	2
Dicalcium phosphate	1.15
Vitamin and trace mineral premix ^1^	0.9
Nutrients, % of DM	
DM, %	89.3
CP, %	22.13
Crude fat, %	4.32
NDF, %	17.14
ADF, %	6.62
Ca, %	1.07
P, %	0.56
ME, Mcal/kg	2.83

DM: dry matter; CP: crude protein; NDF: neutral detergent fiber; ADF: acid detergent fiber; ME: metabolizable energy. ^1^ Premix contained (mg/kg): vitamin A, 4035; vitamin D, 1740; vitamin E, 39; Fe, 18; Zn, 37; Cu, 10.6; Mn, 15.3; Co, 0.12; I, 0.47; and Se, 0.35.
